# The Role of Bioactive Compounds in Human Health and Diseases (2nd Edition)

**DOI:** 10.3390/nu17142279

**Published:** 2025-07-10

**Authors:** Jacqueline I. Alvarez-Leite

**Affiliations:** Departamento de Bioquímica e Imunologia, Universidade Federal de Minas Gerais, Avenida Antônio Carlos 6627, Belo Horizonte 31270-901, Minas Gerais, Brazil; alvarez@ufmg.br

The increasing recognition of bioactive compounds as influential agents in human health has ushered in a new era in nutrition science. These naturally occurring molecules—present in a wide variety of plant- and animal-based foods—are now at the forefront of the discussions surrounding preventative nutrition, functional foods, and integrative therapeutic strategies. Mounting evidence highlights their capacity to modulate critical biological processes, offering potential benefits in mitigating the progression of chronic non-communicable diseases (NCDs), such as obesity, type 2 diabetes, cardiovascular diseases, intestinal diseases, cancer, and neurologic conditions [1,2]. This Special Issue of Nutrients brings together original research and reviews that delve into the uses of bioactive compounds across diverse pathological settings. The works featured herein reflect the interdisciplinary momentum surrounding this field, bridging molecular nutrition, pharmacology, immunology, and clinical practice.

Shimizu et al. (contribution 1) conducted a randomized, double-blind, placebo-controlled clinical trial titled “Preliminary Data on the Senolytic Effects of Agrimonia pilosa Ledeb. Extract Containing Agrimols for Immunosenescence in Middle-Aged Humans”. Agrimonia pilosa Ledeb., or Pilosa Cocklebur, is a plant of the family Rosaceae used in Chinese medicine that contains bioactive compounds, such as phloroglucinols, flavonoids, tannins, and phenols, among others. The study assessed whether an extract standardized in agrimols (APE) could reduce senescent CD8^+^ T cells in adults aged 40–59. While the primary outcomes were not statistically significant across the full cohort after eight weeks of supplementation, exploratory analysis revealed promising immunomodulatory effects in male participants, specifically, reductions in senescent CD8^+^ cells, increases in naïve T cells, and declines in effector memory populations. These findings, though preliminary, underscore the therapeutic potential of APE as a nutritional senolytic agent and reinforce the importance of bioactive compounds in mitigating age-related immune dysfunction.

Ma et al. (contribution 2) contributed a compelling in vitro study investigating metabolic regulation in a HepG2 steatosis model mimicking metabolic dysfunction-associated steatotic liver disease (MASLD). By tracing carbon flux using ^13C-labeled glucose, the authors evaluated the effects of several flavonoids—naringenin, morin, silibinin, and topiramate—on key metabolic pathways. Silibinin and topiramate significantly reduced lipid accumulation and suppressed de novo palmitate synthesis, potentially via the modulation of the pyruvate dehydrogenase (PDH) pathway. In contrast, morin enhanced lipogenesis and inhibited ribose synthesis. The oleic acid-induced model demonstrated that glucose utilization favored lipid storage at the expense of ribose production and fatty acid desaturation. Given that the compound concentrations were aligned with human exposure levels, these metabolic modulations may reflect physiologically relevant outcomes. Nonetheless, future preclinical and clinical research is warranted to explore therapeutic windows, treatment duration, and stage-specific efficacy across MASLD and its progression to MASH.

This Special Issue also welcomes innovative contributions that bridge traditional herbal wisdom with contemporary pharmacological insight. In the study by Budriesi et al. (contribution 3), the authors present HEMEO, a composite formulation composed of herbal extracts (*Asparagus racemosus*, *Tabebuia avellanedae*, and *Glycyrrhiza glabra*) and essential oils (*Foeniculum vulgare*, *Mentha piperita*, and *Pimpinella anisum*), each rich in bioactive constituents like saponins, flavonoids, glycyrrhizin, menthol, and anethole. Ex vivo analysis using isolated guinea pig tissues revealed that HEMEO selectively modulates ileal and colonic motility, with potential relevance to functional gastrointestinal disorders. Moreover, HEMEO showed antibacterial activity against *Helicobacter pylori*, and antioxidant potential, suggesting its multifaceted role as a gastrointestinal health supplement. While the individual ingredients are pharmacologically well characterized, their synergistic combination opens up new avenues for translational research—particularly in the management of motility disturbances, inflammation, and microbial imbalance.

In a complementary direction, Ávila et al. (contribution 4) explored the cardiometabolic benefits of capsaicin, a pungent alkaloid derived from chili peppers. Utilizing an ApoE-deficient mouse model, the authors demonstrated that capsaicin supplementation attenuates atherosclerotic lesions, improves lipid profiles, and reduces systemic inflammation. Mechanistic insights revealed the modulation of cholesterol transport, marked by decreased foam cell formation through the downregulation of scavenger receptor A and upregulation of ABCA1-mediated efflux. These effects were largely dependent on TRPV1 receptor activation, with a complex interaction involving PPARγ. The study offers preclinical support for capsaicin’s anti-atherogenic potential and illustrates the broader theme of dietary compounds acting on nuclear and membrane-bound signaling pathways to influence chronic disease outcomes.

To further enrich this editorial, Lee et al. (contribution 5) published their study, which explores the immunomodulatory capacity of *Euglena gracilis*, a unicellular alga rich in β-1,3-glucan paramylon. Using an in vitro model and a cyclophosphamide-induced immunosuppressed mouse model, supplementation with *E. gracilis* powder restored innate and adaptive immune markers, reversing weight loss, normalizing splenic lymphocyte profiles, and increasing pro-inflammatory cytokines and serum IgM levels. Notably, the hepatic expression of dectin-1—a key receptor in β-glucan-mediated immune activation—was upregulated. These findings highlight *E. gracilis* as a promising natural immunostimulant, particularly under immunocompromised conditions.

Yimam et al. (contribution 6) further addressed immune restoration using UP446, a standardized bioflavonoid composition derived from *Scutellaria baicalensis* and *Acacia catechu*. In mouse models of oxidative stress-accelerated immunosenescence and chemically induced immune suppression, the oral administration of UP446 enhanced NK and T cell responses, antioxidant capacity, and thymic integrity, while suppressing NFκB and restoring IgA and IgG levels. These findings support the potential of UP446 in preserving respiratory and systemic immune resilience, particularly in aging or environmentally stressed populations.

Also featured is the study by Shin et al. (contribution 7), which evaluated the antihypertensive effects of *Lindera erythrocarpa*, leaf ethanolic extract (LEL). Vascular reactivity and in vivo blood pressure assessments revealed that LEL exerts vasodilatory action via the activation of NO–cGMP signaling, calcium channel blockade, and potassium channel opening, coupled with angiotensin II inhibition. Oral dosing reduced systolic and diastolic pressures in hypertensive rats, pointing to LEL’s potential as a multi-target natural therapy for hypertension, pending further safety and mechanistic studies.

Finally, Gonda et al. (contribution 8) addressed an emerging post-pandemic concern: diabetes onset in elderly individuals following COVID-19 infection. In patients aged 80 years and older, those who received *Ficus pumila* L. extract—a traditional Okinawan remedy—showed improved HOMA-β and HOMA-IR indices, indicating an enhanced insulin secretory function and reduced resistance. This study opens up new avenues for the use of plant-based interventions in supporting glycemic control in vulnerable, post-COVID populations.

Altogether, this Special Issue of Nutrients presents a multidimensional exploration of how bioactive compounds interact with key physiological systems, from immunity and metabolism to vascular and endocrine health. The breadth of therapeutic targets and mechanistic insights represented here underscores the role of dietary bioactives as more than just supportive agents; they are foundational tools in the design of personalized, preventative, and integrative health strategies.
